# Unraveling the Burden of Viral and Bacterial Central Nervous System Infections: A Two-Year Retrospective Study

**DOI:** 10.3390/diagnostics15212699

**Published:** 2025-10-24

**Authors:** Nabeel Alzahrani, Ahmed Alshehri, Ali Alshehri, Sameera Al Johani

**Affiliations:** 1 Department of Clinical Laboratory Sciences, College of Applied Medical Sciences, King Saud bin Abdulaziz University for Health Sciences, Riyadh 11481, Saudi Arabia; 2 King Abdullah International Medical Research Center, Riyadh 11481, Saudi Arabia; 3 Division of Microbiology, Department of Pathology and Laboratory Medicine, Ministry of the National Guard Health Affairs, Riyadh 11426, Saudi Arabia

**Keywords:** central nervous system, bacterial meningitis, viral infections, seasonality, aseptic meningitis

## Abstract

**Background/Objectives:** Central nervous system (CNS) infections remain a significant public health challenge and require rapid and accurate diagnosis to guide clinical management. Although the incidence of bacterial meningitis has declined owing to widespread vaccination, viral etiologies continue to dominate CNS infections. The aim of this study was to assess the epidemiological trends, age distribution, and seasonality of CNS infections using multiplex PCR. **Methods:** A retrospective analysis was conducted on cerebrospinal fluid (CSF) samples collected between January 2021 and December 2022 from patients with CNS infections at King Abdulaziz Medical City. A BioFire FilmArray Meningitis/Encephalitis (ME) panel was used to detect pathogens. Patient demographics, pathogen distribution, and seasonal trends were analyzed. **Results:** A total of 2460 CSF samples were tested, of which 130 (5%) were positive for at least one pathogen. Viral pathogens accounted for 82.3% of the infections, with human herpesvirus-6 (HHV-6) (31%) and enterovirus (EV) (20%) being the most common. Bacterial pathogens represented 17.7% of the cases, with *Streptococcus pneumoniae* (6%) and *Escherichia coli K1* (5%) being the predominant bacterial agents. The highest infection burden was observed in infants aged 0–6 months, with a marked male predominance. Seasonal analysis revealed multiple peaks in viral infections, particularly of HHV-6 and EVs, whereas bacterial infections were sporadic, with *Streptococcus agalactiae* and *Streptococcus pneumoniae* peaking in October and November. **Conclusions:** Viral infections, particularly HHV-6 and EVs, dominated CNS infections, with distinct seasonal and age-related variations. These findings underscore the value of multiplex PCR in improving the rapid diagnosis of CNS infections and aiding in timely treatment and antimicrobial stewardship.

## 1. Introduction

Central nervous system (CNS) infections are critical medical emergencies that require immediate and accurate diagnosis and treatment to prevent severe morbidity and mortality. These infections, classified as meningitis, encephalitis, and meningoencephalitis, are caused by various infectious agents, including bacteria, viruses, fungi, and protozoa [1]. The introduction of vaccines has significantly reduced the incidence of bacterial meningitis, shifting the diagnostic focus towards identifying the viral causes. Before the widespread adoption of the MMR vaccine, mumps was the leading cause of viral meningitis. CNS infections caused by viruses such as enteroviruses (EVs) and herpesviruses are among the leading causes of meningitis worldwide, with an estimated increase in incidence from 2.5 million in 1990 to 2.82 million in 2016 [2,3,4].

Although advancements in molecular diagnostics have improved our ability to identify infectious agents, a significant proportion of aseptic meningitis cases, a common form of meningitis, remain challenging to diagnose, with etiologies that are often not identified [5]. The diagnostic challenge is compounded by the diverse etiology of CNS infections, which include various viruses, bacteria, fungi, and protozoa, often overlapping clinical presentations [6]. This ambiguity in identifying the exact agent contributes to underreported cases and affects the quality of patient care.

Individuals with suspected CNS infections can benefit from improved clinical care when pathogens in the cerebrospinal fluid (CSF) are quickly and accurately identified [7,8,9]. However, the traditional approach, which involves culture and pathogen-specific polymerase chain reaction (PCR) testing of the CSF guided by clinical suspicion, has a slow turnaround time and low diagnostic yield. Therefore, effective therapies cannot be administered on time with the indiscriminate use of empirical broad-spectrum antibiotics [10,11,12].

Recent studies using multiplex PCR identified a range of viral agents responsible for CNS infections. In a study conducted in Ankara, Turkey, using a combination of nucleic acid-based assays, herpes viruses and EVs were identified as the primary causes of CNS infections in children [13]. Similarly, a study from Riyadh, Saudi Arabia, reported EV, and human herpesvirus 6 (HHV-6) as common viral etiologies in aseptic meningitis cases, highlighting the predominance of viral infections in males and the pediatric age group [14].

The reliance on advanced molecular diagnostic techniques, such as multiplex PCR panels, offers an unparalleled opportunity to enhance our understanding of the etiology of CNS infection. These tools enable the rapid identification of various viral pathogens directly from CSF specimens, facilitating timely and appropriate clinical management [9,15,16].

The aim of this study was to investigate the ongoing evolution of CNS infection etiology. Furthermore, we examined the distribution of CNS infections among different age groups and the seasonality of these infections. Understanding the prevalence and distribution of pathogens that cause CNS infections is crucial for developing targeted interventions, optimizing patient outcomes, and guiding public health strategies. In contrast to previous regional reports that focused primarily on viral meningitis, this study provides a more comprehensive overview encompassing both bacterial and viral CNS infections over a recent post-COVID-19 period, including an analysis of demographic patterns, seasonal trends, and CSF biochemical correlations. Understanding the prevalence and distribution of pathogens that cause CNS infections is crucial for developing targeted interventions, optimizing patient outcomes, and guiding public health strategies.

## 2. Materials and Methods

King Abdulaziz Medical City (KAMC), a tertiary healthcare facility under the Ministry of National Guard Health Affairs, is a 2500-bed advanced hospital located in Riyadh, Saudi Arabia. In this retrospective study, CSF samples were obtained as part of the clinical evaluation for suspected CNS infection between January 2021 and December 2022. In pediatric patients, lumbar puncture was performed in those presenting with signs or symptoms suggestive of meningitis, or in neonates (<28 days) with fever or other indications of sepsis, in accordance with American Academy of Pediatrics (AAP) guidelines [17]. Testing was performed using the BioFire FilmArray Meningitis/Encephalitis (ME) Panel (BioFire Diagnostics, Salt Lake City, UT, USA) on the BioFire Torch System according to the manufacturer’s instructions.

The BioFire ME Panel is a multiplex PCR-based assay designed to detect and differentiate nucleic acids from a comprehensive set of bacterial, viral, and fungal pathogens associated with meningitis and encephalitis. The panel targeted the following pathogens: *Escherichia coli* K1, *Haemophilus influenzae*, *Listeria monocytogenes*, *Neisseria meningitidis*, *Streptococcus agalactiae*, *Streptococcus pneumoniae*, Cytomegalovirus (CMV), EV, Herpes Simplex Virus 1 (HSV-1), Herpes Simplex Virus 2 (HSV-2), Human Herpesvirus-6 (HHV-6), Human Parechovirus (HPeV), Varicella-Zoster Virus (VZV), and *Cryptococcus neoformans*/*gattii*.

Multiple studies have evaluated the performance of the BioFire ME Panel, demonstrating its high sensitivity and specificity. According to Trujillo-Gomez et al. [18], the panel had a sensitivity range of 88–92% and a specificity exceeding 99% for most targeted pathogens. The assay design incorporated specific primers and probes to minimize cross-reactivity and ensure accurate pathogen identification. Independent assessments, such as those conducted by Messacar et al. [9], further support the reliability of this assay for clinical diagnostics.

Because our dataset was limited to CSF results without systematic extra-CNS testing and with incomplete clinical annotations, most cases could not be robustly classified and are reported without primary/secondary stratification. To identify and characterize these cases, laboratory records from KAMC were reviewed to identify all CSF samples tested during the study period. Data on patient demographics, including age and sex, detected pathogens, and seasonal trends were analyzed. A total of 2460 CSF specimens were included in the study, with some samples originating from the follow-up testing of the same patient. For demographic and pathogen distribution analyses, only unique patient records corresponding to positive cases were included, ensuring that each patient was counted once. Only one patient exhibited co-detection of two pathogens. Ethical approval for this study was obtained from the Institutional Review Board (IRB) of King Abdullah International Medical Research Center (KAIMRC).

Descriptive statistics were used to summarize patient demographics, pathogen distribution, and CSF laboratory parameters. Continuous variables were presented as medians with interquartile ranges (IQRs), and categorical variables as counts and percentages. Comparisons between bacterial and viral infection groups were performed using the Mann–Whitney U test for continuous variables. A *p*-value < 0.05 was considered statistically significant. All statistical analyses were conducted using IBM SPSS Statistics (Version 16, IBM Corp., Armonk, NY, USA). Data were visualized using Microsoft Excel (Microsoft Corporation, Redmond, WA, USA) to generate pie charts. Trend analysis and additional visualization were performed using the Power BI software (October 2024 release; Microsoft Corporation, Redmond, WA, USA).

## 3. Results

In total, 2460 CSF samples were tested from 2021 to 2022. Of these, 130 (5%) tested positive for at least one pathogen. Among the 130 positive samples, only one case (0.8%) demonstrated co-infection with two pathogens, whereas all other cases were single-pathogen infections. The demographic distribution of the patients (Table 1) revealed a predominance of pediatric patients, comprising 73% of the study population. Regarding sex distribution, 63% (*n* = 82) were male, and 37% (*n* = 48) were female. Most samples (82.3%) were positive for viral targets, while the remaining samples (17.7%) were positive for bacterial targets (Table 1). The sole fungal pathogen in the ME panel, *Cryptococcus neoformans*/*gattii* was not detected in any of the samples. Three deaths (2.3%) were recorded among the positive cases, all of which occurred in patients with bacterial meningitis.

As presented in Table 1, bacterial infections demonstrated significantly higher CSF protein concentrations (*p* < 0.001) and lower glucose levels (*p* = 0.0004) compared with viral infections. The total nucleated cell count was also significantly higher in bacterial cases (*p* < 0.001). In contrast, viral infections showed a higher proportion of lymphocytes (*p* = 0.0003) and a lower proportion of segmented neutrophils (*p* = 0.0003). CSF red blood cell counts varied widely between groups and did not show a significant difference (*p* = 0.263).

These findings indicate that bacterial meningitis cases were associated with elevated protein, reduced glucose, and a predominance of neutrophils, whereas viral infections showed higher glucose and lymphocytic predominance, consistent with classical CSF profiles.

### 3.1. Etiology of CNS Infections

**Figure 1:**
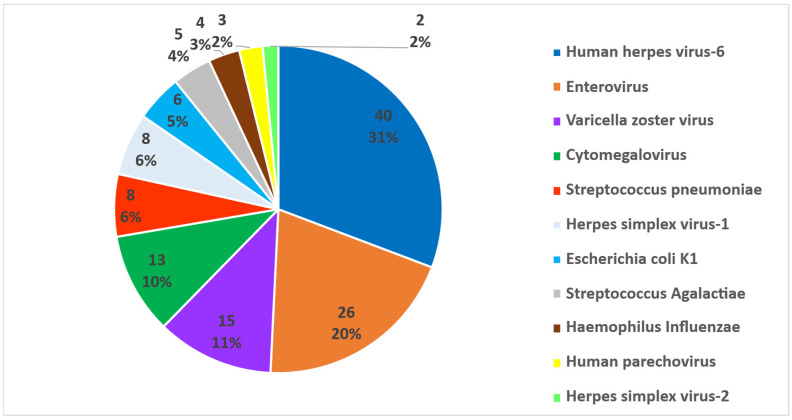
Distribution of pathogens detected by multiplex PCR in cerebrospinal fluid (CSF) samples.

In total, 130 positive cases with a diverse range of viral and bacterial pathogens were identified. Human Herpesvirus-6 (HHV-6) was the most frequently detected pathogen, accounting for 40 cases (31%), highlighting its prominence in the tested population. Enterovirus (EV) was the second most common causative agent (*n* = 26, 20%), followed by Varicella Zoster Virus VZV (*n* = 15, 12%) and cytomegalovirus (CMV) (*n* = 13, 10%). Bacterial pathogens were less prevalent but were still notable. *S. pneumoniae* and Herpes Simplex Virus-1 (HSV-1) were detected in equal numbers (*n* = 8, 6% each), and *E. coli* K1 (*n* = 6, 5%) and *Streptococcus agalactiae* (*n* = 5, 4%) were also identified. *H. influenzae* (*n* = 4, 3%), human parechovirus (HPeV) (*n* = 3, 2%), and Herpes Simplex Virus-2 (HSV-2; *n* = 2, 2%) had the lowest detection rates (Figure 1). The predominance of viral pathogens over bacterial agents suggested a primary viral etiology in the tested cohort, with HHV-6 having the highest detection rate (Figure 1). Bacterial pathogens such as *Neisseria meningitidis*, *Listeria monocytogenes*, and the fungus *Cryptococcus* were not detected in our cohort. The significant prevalence of EV and CMV underscores their roles in the study population. These findings highlighted the importance of continued surveillance to better understand the epidemiological landscape of these infections.

### 3.2. Distribution of CSF Infections by Age Group

Viral pathogens overwhelmingly predominated in the 0–6 month age group. Human Herpesvirus 6 (HHV-6) was the most common pathogen in young infants (*n* = 24) and accounted for the largest number of positive cases (far exceeding that of any other agent in this age group). EV was the second most common etiology in 0–6 month-old infants (*n* = 9), and HHV-6 and EV were found in most of the positive samples in this age category. In comparison, bacterial pathogens (such as *E. coli* K1 or group B *Streptococcus*) were detected in only a minority of infants, indicating a much lower burden than viruses, even in this high-risk age range.

Beyond infancy, the data shows a clear shift in the predominance of pathogens. During childhood, EVs remain a leading cause of CNS infection, notably persisting as the predominant pathogen in school-aged children, whereas HHV-6 detection declines sharply after 6 months of age. Viral etiologies continue to dominate in older children and adults, with herpes viruses becoming the most prevalent in adults. Herpes simplex virus (HSV-1) has emerged as the leading cause of PCR-positive meningoencephalitis in adults, and VZV has become a notable contributor in older adults. In contrast, bacterial positives were limited beyond infancy; only sporadic cases (e.g., a small number of meningococcal or pneumococcal infections in adolescents and adults) were identified. This age-related pattern highlighted the transition from a mixed neonatal disease profile to one dominated by viral pathogens in older children and adults, with a marked reduction in bacterial CNS infections after the first few months of life.

**Figure 2:**
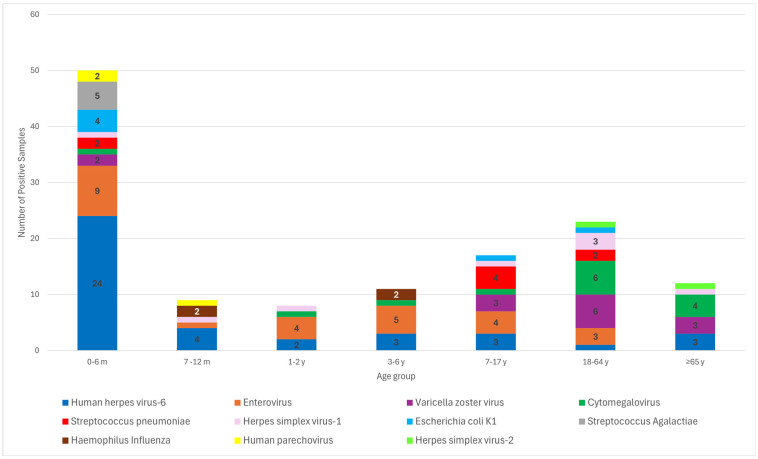
Age distribution of pathogens detected by multiplex PCR in cerebrospinal fluid (CSF) samples.

The number of positive cases decreased as age increased. However, infections persisted in all age groups. In the 7–12 month and 1–2 year groups, infections were more evenly distributed, with EVs, VZV, and CMV being the most commonly detected pathogens. As expected, all *S. agalactiae* cases were exclusive to infants aged 0–6 months. There was a noticeable increase in the diversity of the detected pathogens among older children (3–6 and 7–17 years old), with *S. pneumoniae* and *E. coli* K1 appearing more frequently. This shift suggested an increasing exposure to bacterial infections as children grow older. The 18–64 year age group exhibited the second-highest overall burden of infection, with a more balanced distribution between viral and bacterial pathogens, including VZV, CMV, and *S. pneumoniae*. In contrast, the older adult population (≥65 years) demonstrated a moderate burden of infection, although with a slightly higher representation of bacterial pathogens than the younger age groups (Figure 2). These findings indicated a dynamic age-related pattern of infection, where viral pathogens dominate in early life and bacterial pathogens become more prominent in later years. The high prevalence of HHV-6 in infants and the broad diversity of pathogens in older populations underscore the need for targeted surveillance and age-specific management strategies for infectious diseases.

### 3.3. Seasonal Distribution

**Figure 3:**
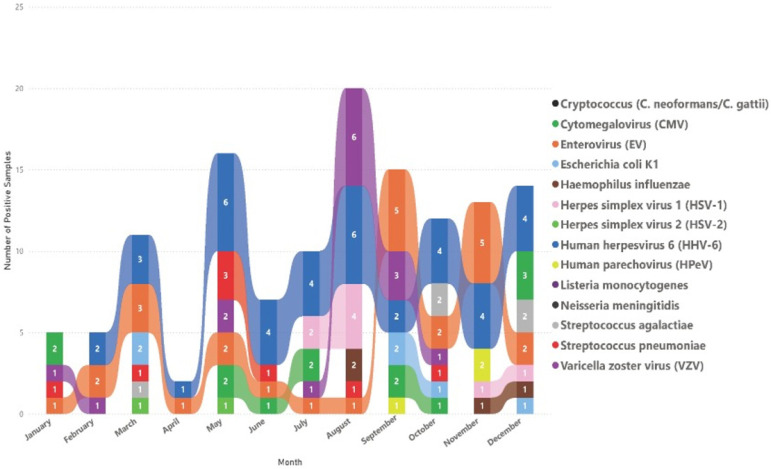
Monthly distribution of pathogens detected in cerebrospinal fluid (CSF) samples by multiplex PCR.

The seasonal distribution of positive samples revealed distinct patterns of infection dynamics throughout the year. HHV-6 and EV were the most frequently detected pathogens. HHV-6 was consistently the most prevalent virus throughout the year, with stable and high detection levels during all months. This continuous detection pattern may reflect frequent reactivation or persistent subclinical infection rather than primary infection outbreaks or seasonal variations. EV was also frequently detected in all months, demonstrating a persistent year-round presence. However, EV showed a clear seasonal increase, with a distinct peak occurring in September and October. Notably, HSV-1 showed increased detection rates, predominantly in late summer (July to August), whereas CMV exhibited slightly elevated detection rates from May to September. *E. coli* K1 and *H. influenzae* exhibited sporadic detection, suggesting a less seasonal pattern, but remained present throughout the year. VZV was most frequently detected in August and September, which coincides with the start of the school year in Saudi Arabia (Figure 3). In summary, the persistent detection of HHV-6 throughout the year may indicate frequent viral reactivation, possibly associated with underlying immune status variations in the studied population. The clear seasonal peak of EVs in September, October, and November aligns with the known patterns of seasonal infection. Understanding these trends could help to inform infection control strategies and targeted surveillance efforts throughout the year.

## 4. Discussion

The findings of this study provide valuable insights into the etiology, demographic distribution, and seasonality of CNS infections diagnosed by multiplex PCR-based testing of CSF samples. Our results emphasize the predominance of viral infections in CNS pathology, particularly highlighting the high prevalence of HHV-6 and EVs while revealing bacterial etiologies and their age-related distribution. These findings are critical for guiding diagnostic approaches and informing clinical decision-making regarding suspected CNS infections. Our study revealed that viral pathogens accounted for 82.3% of the detected cases, with HHV-6 (31%) and EVs (20%) being the most frequently identified. These findings were consistent with global epidemiological data that identify EVs and herpesviruses as leading viral etiologies in CNS infections [19,20]. Previous studies, including those from Turkey and Saudi Arabia, have similarly reported a high burden of EVs and HHV-6 in pediatric cases of aseptic meningitis and meningoencephalitis [13,14]. While HSV-1 is reported as the leading viral cause of meningitis and encephalitis in many adult-dominant cohorts, the predominance of HHV-6 in our study likely reflects the younger age distribution of our population and the inclusion of pediatric cases, where primary HHV-6 infection is more common. The high prevalence of HHV-6 in our cohort, particularly among infants aged 0–6 months, suggested early-life susceptibility to this virus. HHV-6 is known for its widespread seroprevalence, with primary infections often occurring in infancy and frequently manifesting as febrile illnesses or exanthema subitum. However, its role in CNS infections remains unclear and its frequent detection in our study raises questions regarding its clinical significance as a primary pathogen or bystander of neuroinflammatory processes [21,22]. EVs, the second most commonly identified viral pathogen, are well-documented causative agents of viral meningitis, particularly in pediatric populations. Their seasonal peaks, observed in our study from September to November, align closely with previously reported patterns, likely reflecting increased viral transmission in fall associated with school reopening and closer interpersonal interactions among children during this period [23,24]. This seasonality is thought to be driven by EV transmission dynamics, which favor higher temperatures and increased human interactions. Similarly, VZV showed a prominent increase in detection in August and September, coinciding with the start of the school year in Saudi Arabia. This pattern supports previous findings that highlight the role of increased social interaction and community mixing in driving VZV transmission during late summer and early fall, particularly in susceptible pediatric groups [25]. Additionally, this seasonal pattern reinforces the importance of considering the timing of clinical management strategies and preventive interventions, such as targeted awareness campaigns or vaccination programs, to effectively reduce the spread of infection and disease burden.

Although bacterial infections represent only 17.7% of positive cases, they remain a significant concern because of their potentially severe outcomes. In this study, three deaths (2.3%) were recorded, all of which occurred in patients with bacterial meningitis. This mortality rate is lower than the 5–20% reported in several global studies, which may reflect early diagnosis, improved clinical management, and widespread vaccination programs in the studied population [26,27]. *Streptococcus pneumoniae* (6%) and *E. coli* K1 (5%) were among the most frequently detected bacterial pathogens, emphasizing their role in CNS infections. This aligns with global studies in which *S. pneumoniae* was the most common cause of bacterial meningitis in all age groups [28]. As expected, four of the six cases of *E. coli* K1 were in the 0–6 month age group. The reduced burden of bacterial meningitis in our study may reflect the impact of routine vaccination programs, particularly those targeting *H. influenzae* type B (Hib) and *S. pneumoniae*. However, the presence of these bacterial pathogens, particularly in infants and elderly individuals, underscores the need for vigilance in high-risk populations. Notably, we observed a complete lack of *N. meningitidis* cases, which could be a result of COVID-19 preventive measures such as wearing masks, social distancing, and reduced social gatherings during the study period. These public health measures have been associated with a significantly reduced incidence of respiratory pathogens, including *Neisseria meningitidis*. In addition, the absence of N. meningitidis detection in our cohort likely reflects the impact of Saudi Arabia’s national meningococcal vaccination program, which provides the quadrivalent conjugate vaccine (MenACWY) as part of the routine childhood immunization schedule (administered at 9 months and 2 years of age) and mandates vaccination for all pilgrims, healthcare workers, and residents of the holy cities. Although individual vaccination data for our patients were not available, this systemic national vaccination policy ensures high population-level coverage and likely contributes to the near elimination of meningococcal meningitis in our study population. Interestingly, we did not detect *Cryptococcus neoformans*/*gattii*, which are included in the BioFire ME Panel. This could be due to the low prevalence of cryptococcal meningitis in our study population, possibly reflecting regional epidemiological trends, or the absence of high-risk immunocompromised individuals, such as those with HIV/AIDS, from the sample cohort.

Our analysis demonstrated a clear age-related variation in the distribution of CNS infections, with the highest number of cases observed in infants aged 0–6 months. This age group exhibited a striking male predominance (32 males, 18 females), a trend that persisted in several other age categories. The reasons behind this sex disparity remain speculative but could be attributed to differences in immune responses, hormonal influences, or genetic factors affecting susceptibility to infections. Beyond infancy, the frequency of infections declined, but remained notable across all age groups. The 3–6-year and 7–17-year categories displayed an increased diversity of pathogens, with bacterial infections (e.g., *S. pneumoniae* and *H. influenzae*) appearing more frequently. These findings suggested that, as children grow, their exposure to bacterial pathogens increases, possibly due to environmental interactions and changes in immune function. In adults (18–64 years), we observed the second-highest burden of infections, predominantly driven by viral pathogens, especially VZV and EV, with bacterial pathogens comprising a smaller proportion of positive cases.

In contrast, individuals >65 years demonstrated a modest burden of CNS infections exclusively associated with viral pathogens, particularly viruses within the Herpesviridae family, such as the VZV and HHV-6. This pattern aligns with known age-related immunosenescence, which influences susceptibility to viral reactivation, particularly VZV, in older adults [4]. The seasonal distribution of the detected pathogens revealed distinct infection peaks, particularly for viral agents. HHV-6 and EVs exhibited multiple seasonal spikes with notable surges in May, August, and October. These seasonal trends mirror the global patterns of viral CNS infections, where EVs frequently peak in late summer and fall [3]. The environmental and behavioral factors driving these trends may include higher temperatures, increased human mobility, and closer indoor gatherings during certain seasons [29]. In contrast, bacterial infections were sporadically distributed. Notably, only a single case of *Listeria monocytogenes* was identified in October, suggesting sporadic occurrence rather than a clear seasonal trend.

The reliance on multiplex PCR-based assays in this study provided rapid and comprehensive pathogen identification, which is crucial for the timely and targeted management of CNS infections. Traditional diagnostic methods such as culture and pathogen-specific PCR often result in delayed or incomplete diagnoses, leading to the overuse of empirical broad-spectrum antibiotics [9]. Our findings reinforced the utility of multiplex PCR panels such as the BioFire ME Panel in clinical decision-making, particularly for differentiating viruses from bacterial infections and guiding antimicrobial stewardship [10,11,12]. Although multiplex PCR panels offer high sensitivity and specificity, they do not provide information on antimicrobial susceptibility, necessitating the complementary testing of confirmed bacterial cases. Additionally, distinguishing between active viral infections and incidental viral DNA detection remains a challenge, particularly for latent herpes viruses such as HHV-6 and CMV. Future studies should explore the integration of PCR results with host inflammatory markers or metagenomic sequencing to enhance diagnostic accuracy.

Although this study provides valuable insights into the trends in CNS infections, it is limited by its retrospective design and reliance on a single diagnostic platform. The BioFire ME Panel restricts pathogen detection to a predefined panel, potentially overlooking emerging or uncommon CNS pathogens that are not included in the assay. Furthermore, this study did not consider the clinical aspects of the positive cases. Our reliance on CSF-only multiplex PCR precludes confident assignment of primary versus secondary meningitis. Differentiation typically requires paired extra-CNS assays (e.g., blood PCR/serology, lesion swabs), longitudinal viral loads, or detailed clinical context (e.g., rash temporality, neurosurgical history). Additionally, as our study relied solely on CSF molecular detection without paired blood or tissue testing, we cannot fully exclude viral reactivation or bystander detection rather than true neuroinvasive infection in some HHV-6–positive cases. This granularity was beyond the scope of our retrospective dataset and is a key target for future prospective studies. Future studies employing broader metagenomic sequencing approaches could expand our understanding of CNS infection etiologies, particularly in cases where no pathogens have been identified. In addition, although we observed age- and season-related patterns, a longer study duration across multiple years may have provided more robust data on cyclical infection trends. Finally, incorporating clinical outcome data offers a more comprehensive assessment of the impact of different pathogens on disease severity and patient prognosis.

## 5. Conclusions

In conclusion, our study underscores the predominant role of viral pathogens, particularly HHV-6 and EVs, in CNS infections while highlighting the presence of bacterial etiologies in specific age groups. Seasonal variations in the pathogen distribution suggest the need for enhanced surveillance and preventive strategies, particularly during peak infection periods. These findings reinforced the clinical utility of multiplex PCR assays for the rapid diagnosis of CNS infections, ultimately improving patient management and antibiotic stewardship. Future research should focus on expanding diagnostic capabilities, refining the clinical interpretation of PCR results, and integrating multiyear epidemiological data to better understand long-term infection trends.

## Figures and Tables

**Table 1:** Demographic Characteristics, Etiologic Distribution, and Cerebrospinal Fluid (CSF) Indices of Patients with PCR Confirmed Central Nervous System Infections.

Variable	Number (Percentage)		
**Gender**			
Males	82 (63%)		
Females	48 (37%)		
**Age group**			
Adults	35 (27%)		
Pediatrics	95 (73%)		
**Etiology**			
Bacterial	23 (17.7%)		
Viral	107 (82.3%)		
**Death**	3 (2.3%)		
**CSF Indices**			
	**Bacterial (Median, IQR)**	**Viral (Median, IQR)**	***p*-value**
Protein (g/L)	1.65 (1.76)	0.51 (0.73)	<0.001 *
Glucose (mmol/L)	2.0 (1.1)	3.2 (1.2)	0.0004 *
Total Nucleated Cells (cells/µL)	323 (1017)	13 (131)	<0.001 *
Red Blood Cells (cells/µL)	66 (554)	18.5 (1062.5)	0.263
Segmented Neutrophils (%)	62 (61)	12.5 (27.5)	0.0003 *
Lymphocytes (%)	21 (32)	59 (50)	<0.0003 *

Abbreviations: CSF = cerebrospinal fluid; IQR = interquartile range. Data are presented as median (IQR) and *n* (%). For continuous variables, the Mann–Whitney U test was applied to assess differences between bacterial and viral groups. * Statistical significance was defined as *p* < 0.05.

## Data Availability

The datasets presented in this article are not readily available because the data are governed by an institutional policy that prohibits sharing outside the organization. This could be due to intellectual property concerns, strategic interests, or security considerations. Requests to access the datasets should be directed to datamanagment@kaimrc.edu.sa.
